# Distinct fibroblast subpopulations associated with bone, brain or intrapulmonary metastasis in advanced non‐small‐cell lung cancer

**DOI:** 10.1002/ctm2.1605

**Published:** 2024-03-06

**Authors:** Ke Xu, Hao Wang, Yu‐Xia Zou, Huan‐Huan Zhang, Yue‐Nan Wang, Xue‐Ru Ren, Han‐Qi Wang, Ye‐Hong Xu, Jia‐Jun Li, Hao Tang, Cheng He, Song Wei, Tian Tian, Lai‐Lin Li, Hui Zhou, Lin‐Juan Xu, Jing‐Wen Fang, Chuang Guo, Jia‐Xuan Yang, You‐Yang Zhou, Zhi‐Hong Zhang, Yue‐Yin Pan

**Affiliations:** ^1^ Department of Respiratory Oncology Division of Life Sciences and Medicine the First Affiliated Hospital of University of USTC, University of Science and Technology of China Hefei Anhui China; ^2^ Department of Oncology Division of Life Sciences and Medicine the First Affiliated Hospital of USTC, University of Science and Technology of China Hefei Anhui China; ^3^ HanGene Biotech, Xiaoshan Innovation Polis Hangzhou China; ^4^ Department of Rheumatology and Immunology Division of Life Sciences and Medicine the First Affiliated Hospital of USTC, University of Science and Technology of China Hefei Anhui China

**Keywords:** CAF, fibroblast, non‐small‐cell lung cancer, single‐cell RNA‐seq, tumour microenvironment

## Abstract

**Background:**

Bone or brain metastases may develop in 20–40% of individuals with late‐stage non‐small‐cell lung cancer (NSCLC), resulting in a median overall survival of only 4–6 months. However, the primary lung cancer tissue's distinctions between bone, brain and intrapulmonary metastases of NSCLC at the single‐cell level have not been underexplored.

**Methods:**

We conducted a comprehensive analysis of 14 tissue biopsy samples obtained from treatment‐naïve advanced NSCLC patients with bone (*n *= 4), brain (*n *= 6) or intrapulmonary (*n *= 4) metastasis using single‐cell sequencing originating from the lungs. Following quality control and the removal of doublets, a total of 80 084 cells were successfully captured.

**Results:**

The most significant inter‐group differences were observed in the fraction and function of fibroblasts. We identified three distinct cancer‐associated fibroblast (CAF) subpopulations: myofibroblastic CAF (myCAF), inflammatory CAF (iCAF) and antigen‐presenting CAF (apCAF). Notably, apCAF was prevalent in NSCLC with bone metastasis, while iCAF dominated in NSCLC with brain metastasis. Intercellular signalling network analysis revealed that apCAF may play a role in bone metastasis by activating signalling pathways associated with cancer stemness, such as SPP1‐CD44 and SPP1‐PTGER4. Conversely, iCAF was found to promote brain metastasis by activating invasion and metastasis‐related molecules, such as MET hepatocyte growth factor. Furthermore, the interaction between CAFs and tumour cells influenced T‐cell exhaustion and signalling pathways within the tumour microenvironment.

**Conclusions:**

This study unveils the direct interplay between tumour cells and CAFs in NSCLC with bone or brain metastasis and identifies potential therapeutic targets for inhibiting metastasis by disrupting these critical cell–cell interactions.

## Graphical abstract

(1) Three cancer‐associated fibroblast subpopulations, specifically myCAF, iCAF and apCAF, were identified in NSCLC.

(2) apCAF is ascendant in NSCLC with bone metastasis, and iCAF is predominant in NSCLC with brain metastasis.

(3) apCAF might participate in bone metastasis of NSCLC cells via the SPP1‐CD44/SPP1‐PTGER4 signalling pathway. iCAF may also promote brain metastasis though MET‐HGF.

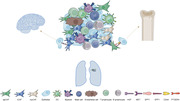

## 1 INTRODUCTION

In 2020, lung cancer recorded a staggering 2.2 million new cases, solidifying its position as the leading cause of cancer‐related deaths worldwide.^1^ Of these cases, approximately 85% fall into the category of non‐small‐cell lung cancer (NSCLC).^2^ Despite considerable therapeutic progress, the 5‐year survival rate for NSCLC remains dishearteningly low, standing at less than 15%. This prognosis often results from the late detection of the disease, with it frequently having already metastasize to the bone, brain or lungs.^3^ Investigations into NSCLC with bone, brain or intrapulmonary metastasis and their associated tumour microenvironment (TME) continue to be conducted.

The NSCLC TME is a complex interplay of tumour cells and non‐tumorigenic cells, including immune cells (T lymphocytes, B lymphocytes, dendritic cells, natural killer cells and macrophages), as well as cancer‐associated fibroblasts (CAFs).^4^ Notably, in metastatic lung adenocarcinoma, single‐cell RNA sequencing has demonstrated that stromal and immune cells within the TME can collaborate to create a pro‐tumoral and immunosuppressive microenvironment.^5^ Activated CD8+ T cells may become exhausted due to the TME's involvement in various inhibitory pathways, including PD‐1, CTLA4, TIGIT and TIM‐3, which are often overexpressed on tumour‐infiltrating lymphocytes.^6^ CAFs within the TME play a role in promoting cancer cell migration, invasion, metastasis and the epithelial‐mesenchymal transition.^7^ For instance, the chemokine CCL5, produced by CAFs, promotes Hepatocellular carcinoma (HCC) metastasis by activating the HIF1/ZEB1 axis.^8^ In addition, CAFs can stimulate cancer cell migration in breast cancer through CXCL12 and TGFβ pathways.^9^ However, most studies investigating the TME of NSCLC have relied on bulk RNA technologies and have not specifically examined the TME of NSCLC metastases originating from the lungs at the single‐cell level. Through single‐cell sequencing, it is imperative to characterise the molecular pathways and intercellular crosstalk in NSCLC with distinct metastatic patterns.

To gain deeper insights into the role of the TME in NSCLC metastasis to the brain, bone and lungs, we performed single‐cell transcriptome sequencing on 14 advanced NSCLC tissue samples. Our analysis revealed substantial cellular and genetic expression heterogeneity in NSCLC with distinct metastatic sites, partly illustrating the mechanisms underlying metastasis to distant locations. Notably, we identified eight fibroblast subpopulations, including myofibroblastic CAF (myCAF), inflammatory CAF (iCAF) and antigen‐presenting CAF (apCAF). Furthermore, the proportions of iCAF and apCAF differed in NSCLC with brain or bone metastasis. Significantly, apCAFs may interact with NSCLC cells through the SPP1‐CD44/SPP1‐PTGER4 axis, promoting bone metastasis in NSCLC. Conversely, iCAFs may interact with NSCLC cells via the MET–HGF signalling pathway, fostering brain metastasis in NSCLC. Moreover, both CAFs and tumour cells exert influence on T‐cell exhaustion and signalling pathways. These findings provide valuable insights into the potential mechanisms underlying NSCLC metastasis and lay the foundation for future therapeutic development.

## 2 MATERIALS AND METHODS

### 2.1 Human specimens

The present study received approval from The Human Investigation and Ethical Committees of Anhui Provincial Cancer Hospital (The First Affiliated Hospital of USTC West District) and was conducted in accordance with the Declaration of Helsinki. The Approval Number is 105/2021. All participants provided written informed consent. Pathologically confirmed cases of NSCLC were observed in 14 individuals (Supplementary Data 1). Among them, approximately 21.5% were female, with an average age of 63.9 years. Fourteen samples were immediately transported to the laboratory for single‐cell isolation. Percutaneous lung biopsies were performed under Computed Tomography (CT) guidance to obtain tumour tissue. The tissues were collected from the following patients: HGF06 (stage IVB), HGF09 (stage IVB), HGF11 (stage IVB), HGFG01 (stage IVB), HGF03 (stage IVB), HGF05 (stage IVB), HGFN01 (stage IVB), HGFN02 (stage IVB), HGF02 (stage IVB), HGF04 (stage IVA), HGF07 (stage IVA), HGF18 (stage IVB), HGF24 (stage IVA) and HGF22 (stage IVA).

### 2.2 Sample collection and preparation

To obtain fresh biopsy samples, we employed CT‐guided lung puncture biopsies. These samples were then frozen in GEXSCOPE Tissue Preservation Solution (Singleron Biotechnologies) and stored at temperatures ranging from 2°C to 8°C until further analysis. Following a single wash in phosphate‐buffered saline (PBS; Thermo Fisher Scientific), the samples were sectioned into small pieces (approximately 1 mm^3^) before being dissociated using the Human Tumor Dissociation Kit (Miltenyi Biotechnologies). Afterwards, the suspensions were filtered through a nylon mesh cell strainer with a mesh size of 70 µm (i‐Quip). The total number of viable cells was determined using a haemocytometer while examining them under a microscope. Subsequently, the suspensions were reconstituted in PBS containing .04% bovine serum albumin (BSA; Sigma‐Aldrich) and stained with .4% trypan blue (Thermo Fisher Scientific).

### 2.3 Single‐cell RNA library preparation and sequencing

Approximately 8000 cells were resuspended and extracted from each sample for library preparation. Single‐cell transcriptome libraries were generated using the single‐cell 3 solution v2 reagent kit (10× Genomics) following the manufacturer's protocols. In brief, the cell suspensions, partitioning oil, reverse transcription master mix and three gel beads were loaded onto a chromium single‐cell chip. Reverse transcription was conducted on single‐cell gel bead‐in‐emulsions (GEMs) utilising a C1000 TouchTM Thermal Cycler (Bio‐Rad). After amplification of cDNA molecules, purification was performed using SPRIselect beads (Beckman Coulter), and libraries were constructed following the manufacturer's instructions. A unique sample index was established during the creation of each sequencing library. Illumina HiSeq X Ten was employed for library sequencing, with each read being 150 base pairs in length.

### 2.4 Single‐cell RNA‐seq data processing

Sequencing data were aligned to the GRCh38 human reference genome downloaded from the 10× Genomics website using Cell Ranger (version 3.1.0) with default parameters. We imported 10× data into Seurat software version 3 with default settings, filtering out genes expressed in fewer than three cells. Quality control measures were applied to exclude cells meeting any of the following criteria: the number of unique genes expressed in these cells was less than 200 or more than 4000, or the proportion of reads mapping to the mitochondrial genome exceeded 25%. Scrublet^10^ was employed with default settings and an automated threshold for doublet score to identify potential doublets. The process included doublet simulation, normalisation, gene filtering, rescaling, Principal component analysis (PCA), doublet‐score calculation, doublet‐score threshold detection and doublet removal. Scrublet operated with its default parameters. Cells labelled as doublets by Scrublet were subsequently removed. Count data were subjected to an SCTransform‐based (Version 1)^11^ normalisation technique to enhance the biological distribution.

### 2.5 Data integration and batch effect correction

To effectively integrate the single‐cell transcriptome data from different samples and mitigate sample‐specific subgroups arising from batch effects, the Seurat integration algorithm was employed. SCTransform normalisation was performed, and the top 3000 most variable genes were selected for each sample in the Seurat integration process. Batch effects among the samples were corrected using the R package Harmony (version 0.1.0).

### 2.6 Data clustering, differential expression analysis and annotation

To perform dimensionality reduction, clustering and identify Differentially expressed genes (DEGs), we utilised the Seurat program. Our approach began with the computation of the PCA matrix from the integrated data, employing the RunPCA function as part of the default Seurat workflow. Subsequently, we constructed a closest neighbour graph using the FindNeighbors function, incorporating a total of 30 nodes. This network included each cell and its closest neighbours. The Louvain algorithm^12^ was then applied to the resulting nearest neighbour graph using the FindClusters function, with a resolution of .1, in order to identify and characterise the clusters. Additionally, we employed the RunUMAP function with 30 different components to visualise the clustering results via Uniform Manifold Approximation and Projection (UMAP).

Differential expression analysis was conducted to identify DEGs within different clusters or metastasis groups. DEGs or cluster‐specific biomarkers were obtained using the FindAllMarkers function with default parameters and the following criteria: an average log2 fold change greater than 2, *p*‐values less than .01 and a minimum fraction of genes expressed in both clusters exceeding .25.

A machine learning algorithm was employed to predict the cell type of each cell. We used data and annotation information from a lung single‐cell RNA sequencing article,^4^ which closely resembled our samples, as a training set. The model was trained and cell annotation was completed using SciBet, a portable and fast single‐cell‐type identifier.^13^ After annotating cell types, we identified eight major cell types based on recognised cell type genes, namely myeloid cells (FCGR3A, LYZ, AIF1), epithelial cells (KRT18, KRT19, EPCAM), fibroblasts (COL1A1, COL1A2, DCN), endothelial cells (PECAM1, FLT1, RAMP2), B lymphocytes (IGHG3, CD79A, MZB1), T lymphocytes (TRAC, CD3D, CD3E), NK cells (GNLY, NKG7, AIF1) and mast cells (MS4A2, KIT, TPSB2). By implementing finer grain in the FindClusters method, we further categorised these primary cell types into 20 sub‐clusters (fibroblasts sub‐cell type: 1000 most variable genes, 15 PCA, resolution of .4; T sub‐cell type: 2000 most variable genes, 30 PCA, resolution of .1).

### 2.7 The validation of CAF subtypes’ annotation

To validate our current CAF subtypes' annotation, we utilised external data for comparison.^14^ Employing the SciBet machine learning prediction model,^13^ we mapped our dataset with cell‐type labels from the reference dataset. Furthermore, we applied multimodal integration analysis (MIA) to assess and compare the similarity between our annotation results and the reference data, accounting for potential biases in cell‐type naming.^15^

MIA employs a hypergeometric test to evaluate the overlap of marker genes between each CAF subtype in our dataset and the reference dataset. Specifically, we considered the top up‐regulated marker genes with adjusted *p* values of .05 or lower and log fold changes greater than .5. These selected genes were used to calculate MIA enrichment scores. This comprehensive analysis aimed to ensure the accuracy and reliability of our CAF subtype annotation by assessing the agreement between our data and the reference dataset.

### 2.8 Gene ontology enrichment analysis

We identified specific DEGs within fibroblast sub‐clusters and the group with brain metastasis in comparison to the group without brain metastasis. Subsequently, we collected enriched Gene Ontology (GO) terms or Kyoto Encyclopedia of Genes and Genomes (KEGG) pathways for each sub‐cluster or group based on these genes. The clusterProfiler package^16^ with default settings was employed for this purpose. An annotation was conducted using the org.Hs.eg.db database to map these DEGs, and the results were visualised using a bar plot.

### 2.9 Gene regulatory network analysis

We employed the SCENIC^17^ algorithm to investigate dynamic regulon activity in four major fibroblast sub‐clusters. The R package GENIE3 was utilised to construct a co‐expression network for identifying transcription factors (TFs) and RcisTarget for DNA motif analysis. AUCell was then used to score each cell. Given the substantial difference in the number of fibroblasts between the brain metastasis group (*n* = 3393) and the no brain metastasis group (*n* = 706), we randomly selected 1000 fibroblasts from the brain metastasis group for calculation. Genes detected in at least 100 fibroblast cells were chosen as input for the SCENIC procedure.

The SCENIC output, containing 218 motifs and its regulon AUC matrix, was processed with the runSCENIC_4_aucell_binarize() function using default parameters to convert the regulon AUC matrix into a binary matrix for further clustering and analysis of differentially expressed TFs. We identified the top 10 differentially expressed TFs for the sub‐cluster in fibroblasts using the Wilcoxon rank sum test with the following criteria: average log2 fold change > .25, *p*‐values < .01, and a minimum fraction of TF‐expressing cells > .7.

### 2.10 Cytokine/receptor interaction analysis

To highlight the significant differences in cell–cell interactions among sub‐clusters of fibroblasts and malignant tumour cells in epithelial cells between the brain metastasis group and the no brain metastasis group, we first utilised the copyKAT^18^ algorithm to predict aneuploid cells among epithelial cells, designating them as malignant tumour cells. We subsequently applied the Cellphone DB(Version 3)^19^ algorithm to single‐cell RNA sequencing profiles from the brain metastasis group and the comparison group to evaluate the effects of interactions between ligands and their corresponding receptors. We focused on cytokine/receptor combinations that were more abundantly expressed in the brain metastasis group compared to the group without brain metastasis.

### 2.11 Cellrank to detect trajectory changes within fibroblast

For the analysis and visualisation of genetic trajectory changes within fibroblast sub‐clusters, we employed the cellrank package.^20^ Using the gene expression matrix and UMAP dimensionality reduction coordinates as input, we explored the potential developmental trajectory of subgroup cells with default parameters.

### 2.12 Immunohistochemistry and evaluation

Paraffin‐embedded lung cancer puncture specimens were sectioned into 4 µm sections for immunohistochemical staining. Haematoxylin and eosin staining was used for initial tissue sectioning. Subsequently, morphologically representative tumour areas were re‐sectioned. The sections were dewaxed with xylene and ethanol, followed by inhibition of endogenous peroxidase with a 3% hydrogen peroxide solution and incubation with 1% BSA at room temperature for 10 min. Incubation at 4°C overnight was carried out with primary antibodies: Anti‐SPP1 monoclonal antibody (1:100; LOT: #39o4953; affinity biosciences), Anti‐AGL (1:100; LOT: BB12027175; affinity biosciences), Anti‐CD44 (1:100; LOT: #37o8571; affinity biosciences), Anti‐cMET (1:100; LOT: #39o4953; affinity biosciences), Anti‐HGF (1:100; LOT: #44o6892; affinity biosciences).

Subsequently, secondary antibody treatment (Horseradish peroxidase anti‐mouse; PR30012, Proteintech) was conducted for 20 min at room temperature. Specific signals were visualised using diaminobenzidine substrate (DAB). Positive immunohistochemical manifestations, characterised by intracellular target proteins stained with brownish‐yellow granules, were counted in one randomly selected high magnification field of view, and the positive rate was calculated. On average, tens of fields of view were counted in each group (Supplementary Data 2).

### 2.13 Statistical analysis

To assess DEGs and TFs between the two groups, we compared the two experimental settings using the Wilcoxon rank sum test, also known as the Mann–Whitney U test. In most cases where it was necessary to test for statistically significant variation in cell‐type fraction between the two scenarios, the Wilcoxon test, a nonparametric test, was employed. *P*‐values were adjusted using the Benjamini–Hochberg method, and the identification of KEGG and GO keywords was also performed using this method.

## 3 RESULTS

### 3.1 Single‐cell analysis uncovered the intertumoral heterogeneity in primary NSCLC with metastasis in bone, brain or intrapulmonary

**FIGURE 1:**
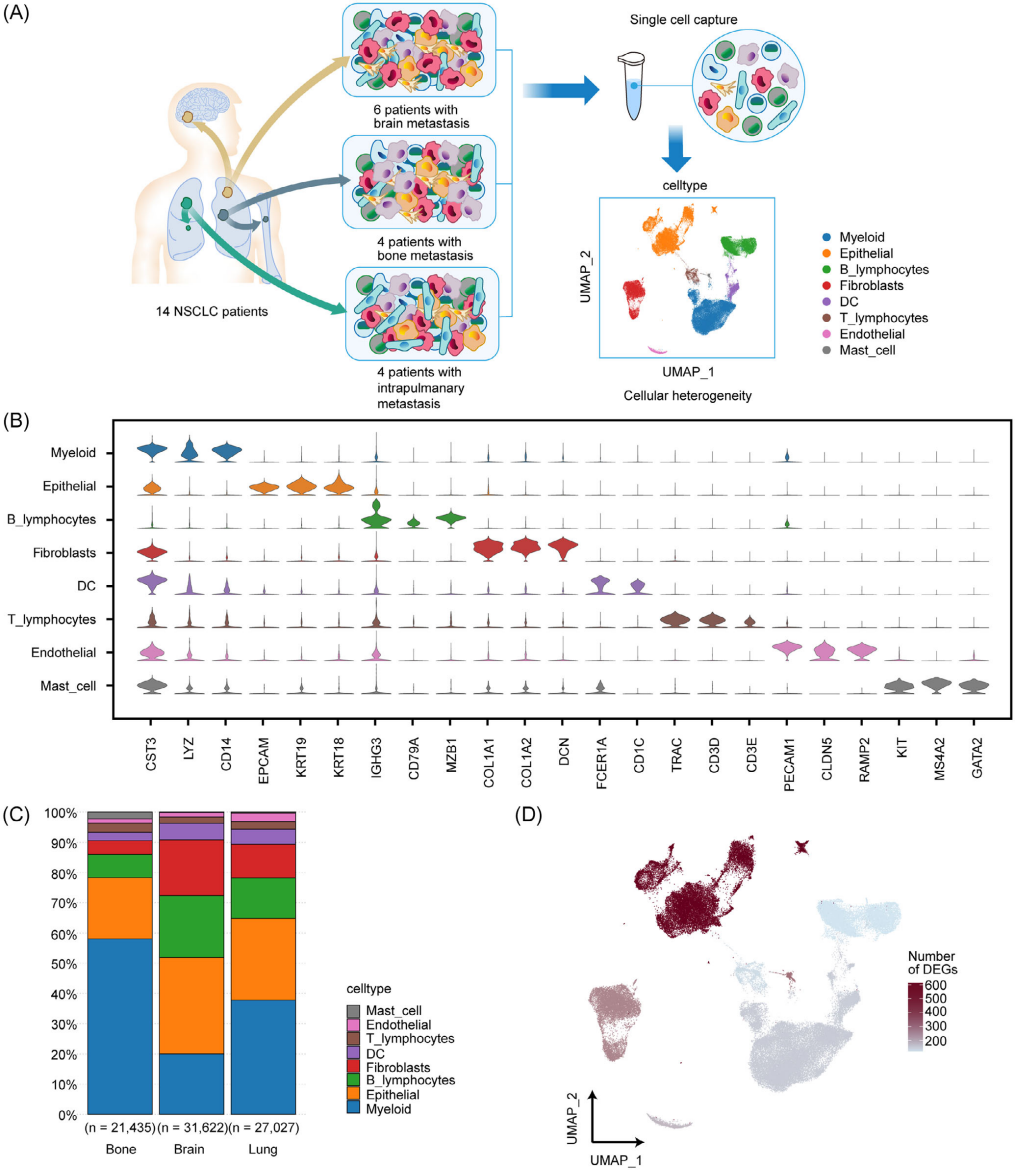
Single‐cell transcriptomic landscape of advanced NSCLC with different metastatic sites originating from the lungs. (A) Schematic graph showing the study design and major cell type annotation. (B) Violin plot showing the expression of representative genes in major cell types. (C) The fraction of major cell types in advanced NSCLC with bone, brain or intrapulmonary metastases. (D) The number of differentially expressed genes (DEGs) among major cell types in advanced NSCLC. (Bone: bone metastasis, Brain: brain metastasis, Lung: intrapulmonary metastasis).

To comprehensively document the cellular and genetic heterogeneity within primary NSCLC tissues with diverse metastatic sites, we conducted single‐cell RNA sequencing on primary tumours from 14 treatment‐naïve NSCLC patients, encompassing bone (*n *= 4), brain (*n *= 6) or intrapulmonary (*n *= 4) metastasis, utilising the 10× Genomics Chromium platform (Figure 1A). We implemented rigorous quality control (QC) to filter out low‐quality cells and removed cell doublets using Scrublet.^10^ Following QC and doublet removal, we successfully captured a total of 80 084 cells, distributed as follows: 21 435 cells from four NSCLC patients with bone metastasis, 3 ,622 cells from six NSCLC patients with brain metastasis and 27 027 cells from four NSCLC patients with intrapulmonary metastasis (Supplementary Figure S1A).

We proceeded to perform unsupervised clustering on the single cells extracted from all 14 specimens, ultimately identifying 14 distinct clusters, which were visualised using the Uniform Manifold Approximation Project (UMAP) (Supplementary Figure S1B). A heatmap in Supplementary Figure S1C illustrates the top three marker genes for each of these 14 clusters.

Subsequently, by leveraging canonical gene markers and the enrichment of differentially expressed genes, we discerned eight major cell types within the dataset: myeloid cells, epithelial cells, B lymphocytes, fibroblasts, dendritic cells, T lymphocytes, endothelial cells and mast cells (Figure 1B). We further investigated the proportions of these major cell types in advanced NSCLC with bone, brain or intrapulmonary metastasis (Figure 1C, Supplementary Figure S1D–E). As previously observed,^10^ although the cellular composition varied, myeloid cells were notably less prevalent in NSCLC patients with brain metastasis compared to those with bone metastasis. Moreover, patients with brain metastasis exhibited a higher proportion of fibroblasts than those with intrapulmonary or bone metastasis, underscoring the cellular composition heterogeneity in primary lung cancer across different metastatic sites (Supplementary Figure S1F).

In addition to these findings, we identified DEGs and quantified the number of DEGs among the three groups, as depicted in Figure 1D. Notably, epithelial cells displayed the highest number of DEGs, followed by fibroblasts, endothelial cells and myeloid cells (Figure 1D, Supplementary Figure S1G). In line with previous research,^21^ our results indicated a substantial degree of genetic heterogeneity in advanced NSCLC based on single‐cell gene expression levels, with carcinoma cells exhibiting a notably high number of DEGs.

### 3.2 Fibroblast cell heterogeneity in primary NSCLC with metastasis in different organs

**FIGURE 2:**
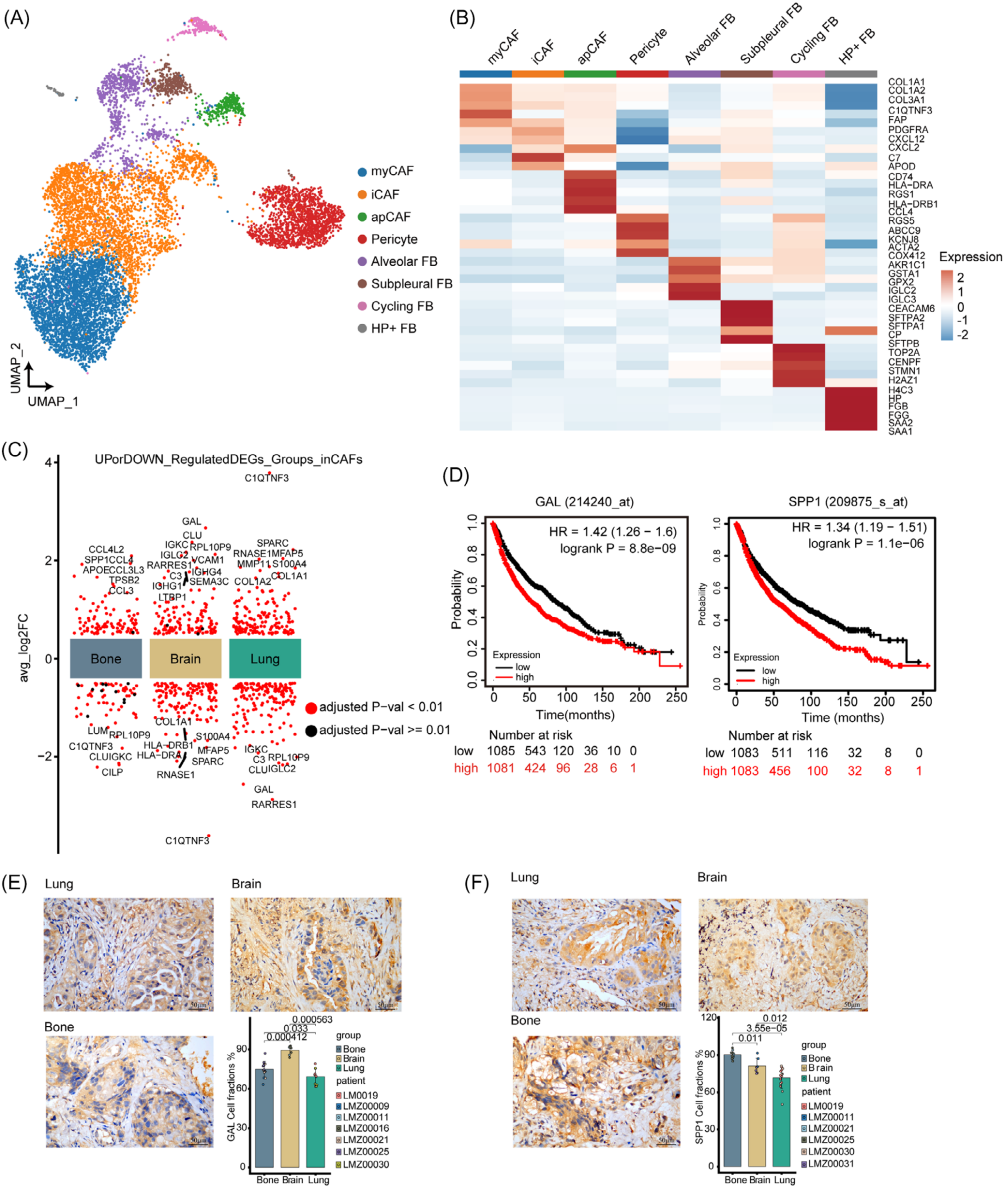
Fibroblast landscape in NSCLC with different metastatic sites originating from the lungs. (A) UMAP plot showing fibroblast subpopulations. (B) Heatmap showing the five most variable genes across each fibroblast subset. (C) DEGs of fibroblasts at different metastatic sites of NSCLC originating from the lungs. (D) Kaplan–Meier curve of overall survival of GAL and SPP1 in NSCLC. (E) Immunohistochemistry analysis of GAL expression in primary NSCLC with intrapulmonary, brain or bone metastases. (F) Immunohistochemistry analysis of SPP1 expression in primary NSCLC with intrapulmonary, brain or bone metastases. (Bone M: bone metastasis, Brain M: brain metastasis, Lung M: intrapulmonary metastasis).

To better understand fibroblast cell diversity and their potential roles in promoting metastasis within advanced NSCLC, we conducted an unsupervised clustering analysis, resulting in the categorisation of fibroblast cells into nine subclusters. We verified the expression purity of fibroblast cell markers such as DCN, basement membrane collagens (COL1A1, COL1A2, and THY1 while also excluding the possibility of doublets. Ultimately, we identified eight fibroblast subgroups, each with distinct characteristic: myCAF, iCAF, apCAF, pericyte, cycling fibroblast, alveolar fibroblast, subpleural fibroblast and HP^+^ fibroblast (Figure 2A). Cluster C0, for instance, expressed high levels of matrix metalloproteinases (MMP13, MMP11) and alpha‐smooth muscle actin (ACTA2), a well‐known myofibroblast marker, signifying the presence of myofibroblasts known to promote tumour progression and angiogenesis.^22^ Consequently, we labelled subcluster C0 as myCAF. Clusters C1 and C3 displayed significant enrichment of apolipoprotein D (APOD) and prostaglandin‐H2 D‐isomerase (PTGDS), pointing to inflammatory features, and were thus designated iCAF. Intriguingly, cluster C6 exhibited high levels of MHC class II family genes (HLA‐DRA, HLA‐DPB1 and HLA‐DPA1), typically restricted to antigen‐presenting cells, leading to the name apCAF. Additionally, cluster C2 displayed high expression of RGS5, a pericyte marker. Cluster C4 exhibited high expression of COL1A1, COL1A2 and DCN, and cluster C8 displayed high levels of SFTPA1, SFTPA2 and SFTPB. Based on the previous studies,^23^ these were classified as Alveolar fibroblasts and Subpleural fibroblasts, respectively. Cluster C8, characterised by high expressions of HP, was labelled as HP^+^ fibroblasts (Figure 2B, Supplementary Figure S2A–B).

We further refined our fibroblast subpopulations using external lung cancer single‐cell data with pre‐existing CAF subtype annotations. Our main objective was to ensure accurate annotations for the three CAF subpopulations of interest. Our results indicated that the majority of myCAFs were correctly annotated as mCAFs. In the iCAF subpopulation, most cells were accurately annotated as iCAFs, although a few were mislabelled as mCAFs. For the apCAF subpopulation, the majority were correctly annotated as apCAFs, with a few minor discrepancies from the expected annotations (Supplementary Figure S2C). To confirm the similarity between our CAF annotation results and the reference subgroups, we conducted a hypergeometric distribution test comparing marker gene enrichment in both datasets. This analysis consistently revealed the highest enrichment scores for myCAF/mCAF, iCAF/iCAF and apCAF/apCAF in both our annotated subpopulations and the reference dataset (Supplementary Figure S2D), substantiating the accuracy of our annotation process.

We also investigated the proportions of fibroblast subtypes in NSCLC with bone, brain or intrapulmonary metastasis (Supplementary Figure S2E). These proportions exhibited substantial variations among the samples (Supplementary Figure S2F). Notably, the proportion of iCAF in NSCLC with brain metastasis exceeded that in NSCLC with bone metastasis. In line with previous reports, iCAFs have been associated with promoting tumour metastasis and organ‐specific metastasis.^24^, ^25^ Conversely, the proportion of apCAF in NSCLC with bone metastasis was significantly higher than in NSCLC with brain metastasis (Supplementary Figure S2G). To delve deeper into the potential association of apCAF, iCAF and myCAF with metastasis, we analysed NSCLC samples from the TCGA database. The results revealed that the gene set scores for apCAF, iCAF and myCAF were all significantly higher in the metastatic group (Supplementary Figure S3A), as were the total top gene set scores for apCAF, iCAF and myCAF (Supplementary Figure S3B). Taken together, our findings suggest that iCAF and apCAF may be involved in NSCLC brain and bone metastasis, respectively.

To explore the functional variations of CAFs in primary NSCLC associated with different metastatic sites, we proceeded to identify the DEGs of CAFs (myCAF, iCAF and apCAF) across various metastatic sites and subsequently conducted an enrichment analysis (Figure 2C, Supplementary Figure S3D). In NSCLC cases with bone metastasis, CAFs exhibited 97 up‐regulated and 62 down‐regulated genes compared to other metastatic sites. The most enriched GO terms included ‘collagen fibril organisation’ and ‘antigen processing and presentation of peptide antigen via MHC class II’. These results align with previous findings, indicating that apCAF‐mediated antigen presentation activates CD4^+^ T cells.^26^ Conversely, in NSCLC with brain metastasis, we observed 133 up‐regulated and 414 down‐regulated genes in CAFs. The most enriched GO terms encompassed ‘lymphocyte‐mediated immunity’ and ‘immunoglobulin‐mediated immune response’. These findings are in concordance with previous research highlighting the ability of iCAFs to secrete inflammatory factors such as IL‐6 and CXCL‐12, which interact with T cells.^24^ Additionally, the expression of galanin (GAL), a protein that modulates the neural niche to favour perineural invasion,^27^ was noted to be highly expressed in NSCLC with brain metastasis (Supplementary Figure S3C). Notably, the survival analysis showed that high expression of GAL was associated with a poor prognosis. These results emphasise the predictive potential of GAL^+^ fibroblasts in NSCLC with brain metastasis. Furthermore, high expression of secreted phosphoprotein 1 (SPP1) also indicated a poor prognosis (Figure 2D).

To corroborate the differential expression of GAL and SPP1 in fibroblasts in NSCLC tissues with distinct metastatic sites, we conducted immunohistochemistry. The results revealed that GAL was most highly expressed in fibroblast of NSCLC tissues with brain metastases, whereas SPP1 was predominantly expressed in fibroblast of NSCLC tissues with bone metastases (Figure 2E‐F).

Subsequently, we delved into the role of TFs in CAFs, as they may be implicated in promoting NSCLC metastasis. TFs and their regulated genes, known as regulons, comprise a complex network that influences cell‐to‐cell variability in cancer.^28^ We employ ‘SCENIC’ analysis to infer the activity of each TF and its associated regulon in myCAF, iCAF and apCAF. By assessing the specificity of regulons, we identified the key regulons for each cell type (Supplementary Figure S3E). For myCAF, the top regulons were TCF12, FOSL2 and CREB3. Notably, CAF TCF12 has been linked to promoting breast cancer growth,^29^ whereas elevated FOSL2 levels in breast CAFs are significantly associated with angiogenesis and clinical progression in breast cancer.^30^ In the case of iCAF, NR2F1, GATA6 and TCG21 were identified as the primary regulons. GATA6 can modulate the chromatin landscape of lung cancer cells to control their proliferation.^31^ For apCAF, SP11, RUNX3 and MAF emerged as the principal regulons. These outcomes point to distinct TF activity and regulons among CAF subtypes that align with their specific functions.

### 3.3 Cancer cell landscape of non‐small‐cell lung cancer

**FIGURE 3:**
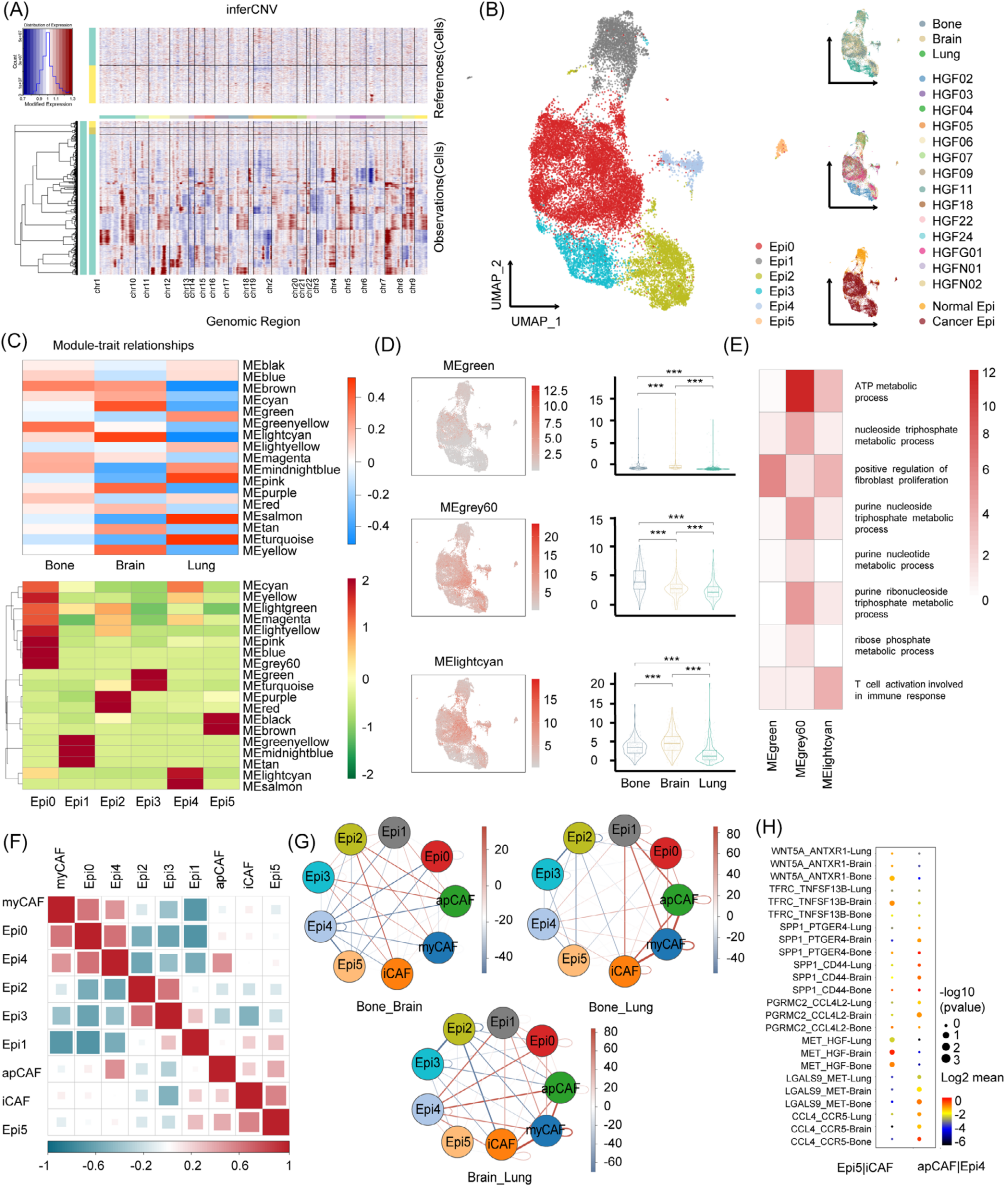
Normal and tumour epithelial cell landscape in NSCLC with different metastatic sites originating from the lungs. (A) Heatmap showing large‐scale CNVs of epithelial cells. Red indicates genomic amplifications and blue reveals genomic deletions. The expression values for B cells and myeloid cells are plotted in the top heatmap, and the epithelial cells are plotted in the bottom heatmap. Genes are ordered from left to right across the chromosomes. (B) UMAP plot of epithelial cells. Data are colour coded by subtypes (left), metastatic sites (upper‐right), patient origins (middle‐right) and tissue origins (lower‐right). (C) Association between the gene modules of epithelial cells and NSCLC with different metastatic sites (top), and association between the gene modules of epithelial cells and subtypes of epithelial cells (bottom). (D) UMAP plot and DEGs of the green module (upper), grey60 module (middle) and light cyan module (lower). Two‐sided unpaired Wilcoxon test was performed to compare between the groups. (E) The GO analysis of the green module, light cyan module and salmon module. (F) Heatmap of cell ratio correlations between CAFs subsets and epithelial subsets. (G) Circos plot showing intercellular interactions between CAFs subsets and epithelial cell subsets in NSCLC with bone, brain or intrapulmonary metastasis. (H) Dot plot showing the mean expression level and percentage of selected interaction pairs involved in bone, brain or intrapulmonary metastasis of NSCLC between epithelial cells and CAFs subtypes. (Bone: bone metastasis, Brain: brain metastasis, Lung: intrapulmonary metastasis).

In the subsequent step, we focused on epithelial cells and distinguished 16 distinct clusters. These epithelial cells displayed considerable variation among samples, consistent with prior observations in related studies (Supplementary Figure S4A). To differentiate malignant from normal epithelial cells, we utilised somatic large‐scale chromosomal CNVs and computed CNV scores in relation to reference normal cells (B cells and myeloid cells) (Figure 3A). Employing the K‐means algorithm, we re‐clustered each epithelial cell based on its infer‐CNV score, which yielded 10 distinct classes (Supplementary Figure S4B). Classes five and six exhibited significantly lower scores than the other classes, thus being categorised as normal epithelial cells (Supplementary Figure S4C–E). Conversely, the remaining groups were identified as cancer cells. To mitigate batch effects among the samples, we applied Harmony correction. This process led to the identification of six distinct clusters (Figure 3B). Ultimately, cluster C1 (Epi1) was designated as normal epithelial cells, while the remaining clusters were characterised as cancer cells. To substantiate this classification, we examined the expression of hallmark canonical cell markers of cancer epithelial cells (EPCAM, SOX4, KRT7, KRT18, KRT19, KRT8) in C1 (normal epithelial cells) and the other clusters (tumour epithelial cells). Notably, the marker genes of cancer epithelial cells exhibited lower expression in C1 (normal epithelial cells) (Supplementary Figure S4F).

Moreover, we ascertained the top 10 DEGs for each subcluster (Supplementary Figure S5A). In line with the previous research, SFTPA1, a marker gene for normal epithelial cells, exhibited high expression in cluster C1 (Epi1).^32^ The proportions of different clusters in primary NSCLC with various metastatic sites demonstrated heterogeneity (Supplementary Figure S5B). Subsequently, we identified the DEGs of epithelial cells in different metastatic sites and conducted an enrichment analysis of these DEGs (Supplementary Figure S5C–D). In NSCLC with bone metastasis, epithelial cells displayed 229 up‐regulated and 136 down‐regulated genes compared to other metastatic sites. The most enriched GO term was the ‘cellular modified amino acid metabolic process’. In NSCLC with brain metastasis, 165 up‐regulated and 162 down‐regulated genes were observed in epithelial cells. The most enriched GO term was the ‘negative regulation of endopeptidase activity’.

To delineate the distinctions in epithelial cells between bone, brain and intrapulmonary metastasis of NSCLC, we employed an unbiased approach to reveal coherent sets of genes (Figure 3C). These gene modules, preferentially co‐expressed by subsets of epithelial cells, were identified through Weighted gene co‐expression network analysis (WGCNA). Four gene modules were identified in all tumour samples, each associated with different biological functions, including cell differentiation, migration and cell cycle regulation. Notably, the green module featured genes involved in the positive regulation of fibroblast proliferation (ESR1, EREG, AGT, BTC), while the light cyan module was enriched for genes associated with T‐cell activation within the immune response context (NFKBIZ, RIPK2, ZC3H12A). These modules were primarily distributed in Epi0/Epi4, closely associated with the brain group. In contrast, the grey60 module, present in Epi0/Epi2, showed enrichment in metabolic‐related signalling pathways and a closer association with the bone group (Figure 3D, E). These recurring gene modules across various samples underscore the heterogeneity among bone, brain and intrapulmonary metastases of NSCLC.

To gain insights into the mechanisms of CAF involvement in primary NSCLC with diverse metastatic sites, we constructed a heatmap illustrating correlations between CAF subtypes and epithelial cell subtypes (Figure 3F). Remarkably, Epi C(4) exhibited a significant association with myCAF and apCAF, while Epi C(5) displayed a strong connection with apCAF and iCAF. These findings suggest that CAFs potentially interact with cancer cells to facilitate NSCLC metastasis. We further deduced putative cell‐to‐cell interactions using CellphoneDB to elucidate intercellular crosstalk between CAFs and epithelial cells in advanced NSCLC (Figure 3G, H). In agreement with our previous observations, apCAF and Epi C(4) in NSCLC with bone metastasis displayed more interactions compared to NSCLC with brain or intrapulmonary metastases. Simultaneously, iCAF and Epi C(5) demonstrated increased interactions in NSCLC with brain metastasis, as opposed to NSCLC with bone or intrapulmonary metastasis.

**FIGURE 4:**
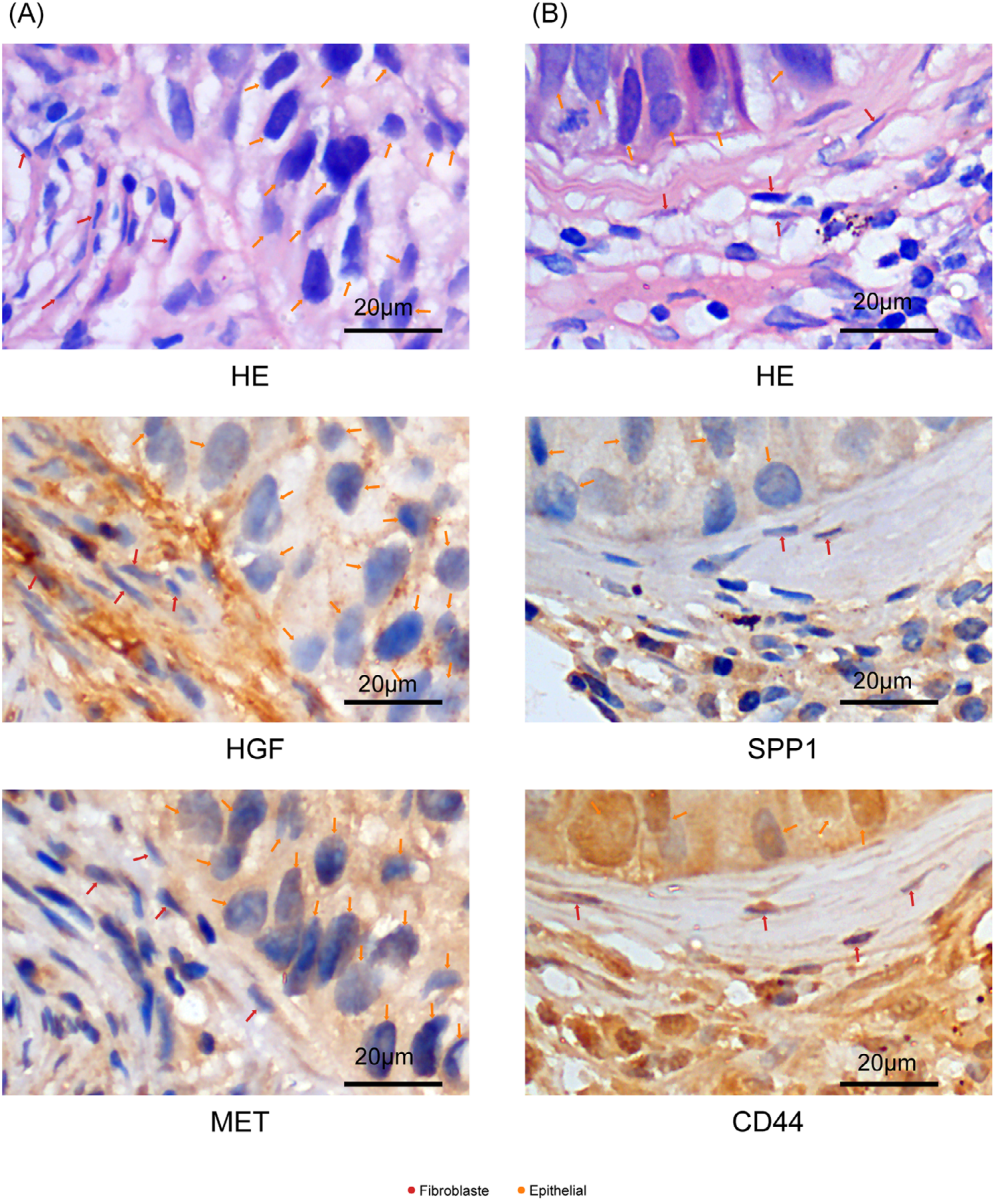
IHC analysis of communication between cancer epithelial cells and fibroblast. (A) IHC detection of continuous tissue sections surrounding MET+ cancer epithelial cells and HGF+ fibroblasts. (B) IHC detection of continuous tissue sections surrounding SPP1+ fibroblast and CD44+ cancer epithelial cells.

To investigate how CAFs engage with cancer cells to promote NSCLC metastasis, we identified crucial receptor‐ligand pairs between CAFs and epithelial cells (Figure 3H). Among these, SPP1 has been implicated in tumour bone metastasis.^33^ Previous studies have predicted strong SPP1–CD44 interactions and SPP1–PTGER4 interactions in hepatocellular carcinoma cells and CAFs.^34^ Furthermore, CAFs can induce pancreatic cancer cell stemness through the SPP1/CD44 axis.^35^ Our findings indicate that SPP1 may mediate crosstalk between apCAF and Epi C(4) through the SPP1‐CD44 and SPP1‐PTGER4 interactions. PTGER4 can stimulate bone resorption, and the PTGER4 antagonist suppresses osteolysis due to bone metastasis of mouse malignant melanoma cells.^36^ Remarkably, the interactions involving SPP1 were significantly enriched in primary tumours with bone metastases (Figure 4B). In addition, hepatocyte growth factor (HGF) appeared to mediate communication between Epi C(5) and iCAF via the MET‐HGF association. CAFs are known to predominantly secrete HGF, and activation of the MET receptor tyrosine kinase can enhance the invasiveness, epithelial‐to‐mesenchymal transition and the activation of multiple oncogenic pathways in breast cancer.^37^ Activation of the MET‐HGF signalling pathway has also been implicated in enhancing pancreatic cancer cell migration and invasion, facilitating cancer cells' ability to invade nerves.^38^ Notably, strong interactions involving MET‐HGF were observed between Epi C(5) and iCAF in NSCLC with brain metastasis. Moreover, our IHC data revealed that MET+ cancer epithelial cells were closely surrounded by HGF+ fibroblasts (Figure 4A), indicating potential communication through the MET‐HGF signalling pathway. Consequently, we propose that iCAFs may interact with NSCLC cells via the MET‐HGF signalling pathway, thereby promoting brain metastasis.

### 3.4 TME in NSCLC with different metastatic sites originating from the lungs

T cells, pivotal for maintaining tissue homeostasis and inflammation regulation in NSCLC, assume a crucial role. Subclustering T cells, as depicted in Supplementary Figure S6A–C, revealed subtypes encompassing regulatory T cells (Treg, FOXP3^+^, IL2RA^+^), CD4^+^ T cells (CD4^+^, CD44^+^), proliferative T cells (MKI67^+^, TOP2A^+^) and CD8^+^ T cells (CD8A^+^, CD8B^+^). Analysing the DEGs of T cells in NSCLC metastases originating from the lungs, we conducted a GO enrichment analysis of these DEGs (Supplementary Figure S6D–E). Our analysis revealed that melanoma inhibitory activity member 3 (MIA3) exhibited heightened expression in T cells within NSCLC brain metastases. MIA3 is known to be involved in tumour cell invasion and migration.^39^ Similarly, CCL20, a crucial chemoattractant linked to inflammatory cell recruitment, displayed overexpression, which can promote lung cancer cell migration, proliferation and CD8^+^ T‐cell recruitment.^40^ Such overexpression was observed in NSCLC with bone metastasis, hinting at its significance. While these findings suggest that MIA3 and CCL20 may be crucial in mechanisms through which T cells promote brain and bone metastasis in lung cancer, additional research is needed for confirmation.

Turning our attention to B cells and myeloid cells in primary NSCLC with different metastatic sites, we examined DEGs and conducted an enrichment analysis (Supplementary Figure S7A–B). Several genes were significantly expressed in B cells of NSCLC with bone metastasis, whereas GADD45A and HSPB1 were highly expressed only in B cells of NSCLC with brain metastasis. GPI, GRI and HSPB1 were highly expressed only in myeloid cells of NSCLC with bone metastasis, while HBEGF, HIF1A, MACF1, MMP9, PTGS2 and BEGFA were highly expressed only in myeloid cells of NSCLC with brain metastasis. The GO enrichment analysis showed that these genes were involved in regulating epithelial cell migration. We speculate that these genes may be essential in B and myeloid cells specific to the metastatic site, implying their role in promoting NSCLC metastasis. However, further studies are required to confirm their precise contributions.

**FIGURE 5:**
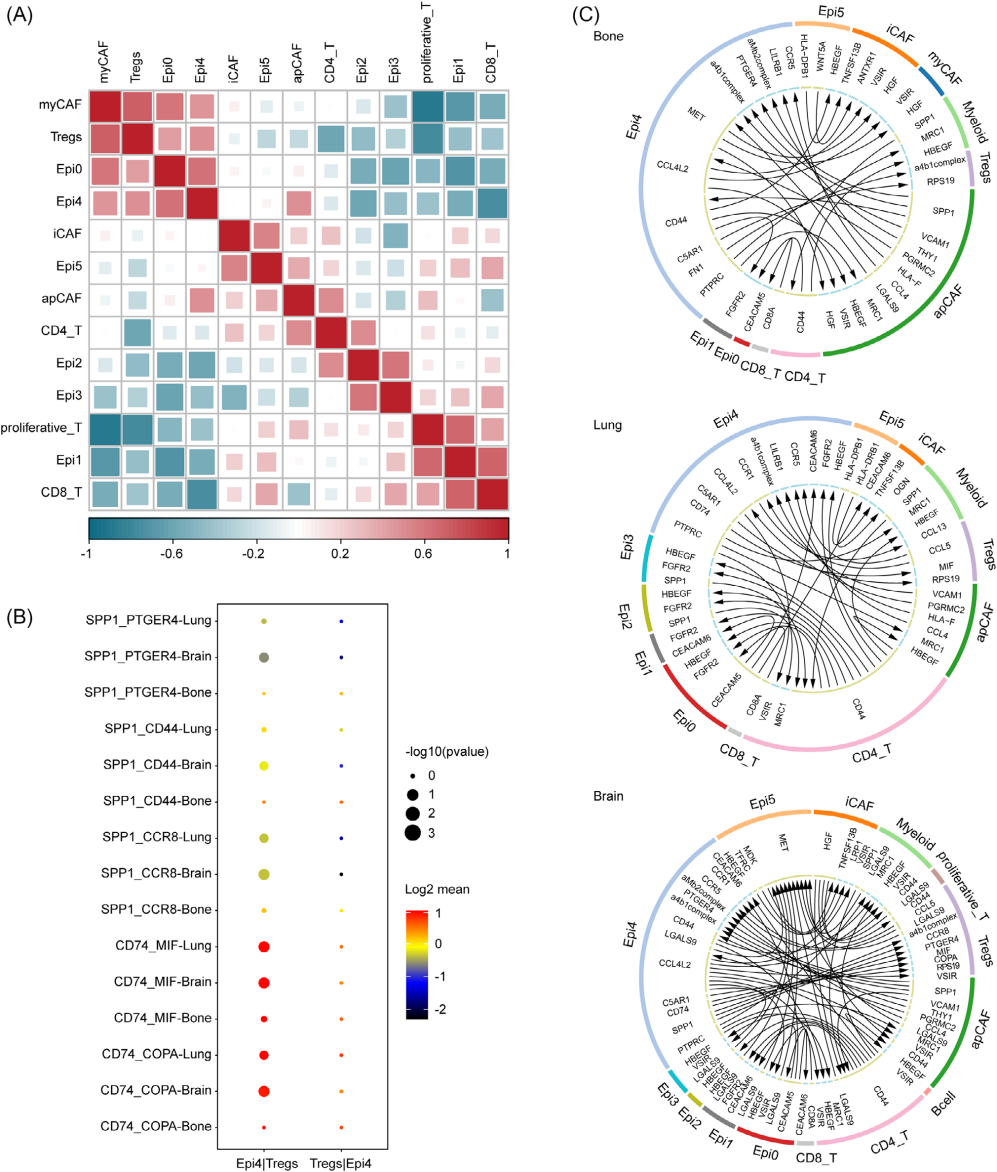
Tumour microenvironment landscape in NSCLC with different metastatic sites originating from the lungs. (A) Heatmap of proportional correlation among subsets of CAFs and epithelial and T cells. (B) Ligand–receptor (L‐R) interaction showing epithelial subsets and T cell subsets involved in bone, brain or intrapulmonary metastasis of NSCLC. (C) Circos plots showing details of L‐R pairs among different cells in NSCLC with bone, brain or intrapulmonary metastasis. (Bone: bone metastasis, Brain: brain metastasis, Lung: intrapulmonary metastasis).

To delve into the mechanisms by which TME cells promote NSCLC metastasis, we constructed a heatmap illustrating the correlations between T‐cell subtypes, epithelial cell subtypes and CAFs (Figure 5A). Notably, the proportion of Tregs exhibited a significant association with Epi C(4) and myCAFs, while the proportion of CD4^+^ T cells was significantly associated with apCAFs. With the assistance of CellphoneDB, we identified crucial ligand–receptor (L‐R) pairs between T cells, CAFs and epithelial cells (Figure 5B). Notably, SPP1 was suggested to mediate the crosstalk between Treg and Epi C(4) via SPP1‐CD44, SPP1‐PTGER4 and SPP1‐CCR8 associations, particularly in NSCLC with brain metastasis. This interaction is of particular interest.

Furthermore, the ligand–receptor interaction CD74‐MIF, which plays a significant role in immunotherapy‐induced hyper‐progressive disease, was also observed between Treg and Epi C(4).^41^ Additionally, the CD52‐SIGLEC10 interaction was noted to suppress T cell activity.^42^ Changes in the interaction between CD4^+^ T cells and apCAFs could be relevant to brain metastasis in NSCLC. The intercellular interactions unveiled the intricate dynamics between immune cells and molecular features that might influence NSCLC metastatic sites (Figure 5C). This suggests a tight link between immune cell dynamics and molecular features that may affect NSCLC metastatic site determination.

## 4 DISCUSSION

Metastatic NSCLC to the bone, brain and lungs presents significant clinical challenges, often associated with a poor prognosis. In this study, we have provided a comprehensive cellular landscape of NSCLC metastases to these different sites. Our findings have revealed distinct tumour cell features and introduced novel CAF subtypes within primary NSCLC, thus shedding light on the potential cues and pathways through which the TME promotes brain or bone metastases in NSCLC. These results lay the groundwork for identifying molecular therapeutic targets for NSCLC with metastases to the brain or bone.

Within the TME, CAFs play a crucial role in cancer progression. Notably, the apCAF subgroup of CAFs stands out due to its significant gene and pathway enrichment related to the MHC class II family. Moreover, our analysis revealed that NSCLC with bone metastasis had a notably higher proportion of apCAFs than NSCLC with brain metastasis. This finding aligns with previous observations of MHC II‐expressing apCAFs in other cancers, such as pancreatic cancer^26^ and oesophageal squamous cell carcinoma.^43^ In oesophageal squamous cell carcinoma, apCAFs modestly expressed the costimulatory genes CD80 and CD86. Interestingly, apCAFs in advanced NSCLC exhibited significantly higher levels of CD74, suggesting variations in their antigen presentation functions across different tumour types. The subtypes of fibroblast are not 100% comparable, although different subtypes of CAFs have been verified in single‐cell studies of different cancer types and this also provides us with more opportunities to explore subtypes of CAF.

Furthermore, our CellphoneDB analysis indicated that SPP1 (osteopontin) mediated the crosstalk between apCAFs and NSCLC cells, particularly through its association with CD44 and PTGER4 in NSCLCs with bone metastases. This interaction mirrors previous findings in hepatocellular carcinoma cells and CAFs, emphasising the role of SPP1‐CD44 and SPP1‐PTGER4 in promoting cancer cell metastasis. These results suggest that the SPP1‐CD44/SPP1‐PTGER4 axis between cancer cells and apCAFs may be instrumental in explaining bone metastasis in NSCLC, in line with prior research highlighting the role of SPP1 in driving metastasis in various cancer types, including colon^44^ and renal cancer.^45^

Previous studies have shown that CAF cells play an essential role in the process of tumour invasion and metastasis. Therefore, we performed a more detailed analysis of the CAF cells. Our results showed that apCAF may play a role in bone metastasis through SPP1‐CD44 and SPP1‐PTGER4. Meanwhile, iCAF was found to promote brain metastasis by MET‐HGF. Consisting with our study, Sun^46^ has proven that CAF could promote tumour development through communicating with other immune cells in lung cancer. CAF has also been shown to be involved in stromal remodelling and epithelial‐mesenchymal transition, which, in turn, promotes tumour metastasis^5^, ^47^ in lung cancer. However, in lung cancer with brain metastases, the proportion of myCAFs were significantly increased.^48^ While in our study, iCAF dominated in NSCLC with brain metastasis. It may be due to the fact that our tissue is derived from primary lung cancer. Our study further enriches the important role that CAF plays in different metastasis sites.

Another noteworthy fibroblast subset is the iCAF, which is characterised by the exclusive expression of inflammatory genes such as Platelet derived growth factor receptor alpha (PDGFRA) and chemokines like CXCL12 and CXCL2. Our findings align with previous research as iCAF subtypes have been identified in various cancers including gastric cancer,^24^ oesophageal squamous cell carcinoma,^49^ pancreatic cancer^50^ and cholangiocarcinoma.^51^ In our study, we also identified APOD and C7 as markers specific to the iCAF population. Intriguingly, the proportion of iCAF in NSCLC with brain metastasis significantly exceeded that in cases with bone metastasis. This observation is in line with the known interaction of iCAF with T cells in gastric cancer where it promotes tumour‐invasive activities by secreting IL‐6 and CXCL12.^24^ Additionally, in cholangiocarcinoma, iCAF has been shown to enhance tumour growth by expressing HGF, which can directly interact with the tumour‐expressed MET receptor.^51^ The consistency of our findings with these studies suggests that iCAFs may interact with NSCLC cells through the MET–HGF signalling pathway to facilitate brain metastasis. However, it is worth noting that a study on pancreatic ductal adenocarcinoma indicated that iCAF was a protective factor associated with a better prognosis,^52^ highlighting the diverse functions of iCAF in different tumour types.

The role of Tregs in cancer progression and metastasis has been the subject of ongoing research. Elevated Treg frequencies have been associated with an increased risk of metastasis in breast, prostate and colon cancer.^53^, ^54^, ^55^, ^56^ Similarly, Tregs have been shown to promote lung cancer development, progression and metastasis via the CXCR4–CXCL12 signalling pathway.^57^, ^58^ Our data reveal a correlation between Tregs and NSCLC metastasis. Notably, our analysis points to SPP1 as a mediator of the crosstalk between Tregs and NSCLC cells through associations with CD44, PTGER4 and CCR8, particularly in NSCLC cases with brain metastasis. This finding suggests that SPP1 may play an essential role in how Tregs promote brain metastasis in NSCLC. However, further experiments are necessary to validate this mechanism.

In summary, this study serves a valuable resource for comprehending the genetic and cellular heterogeneity in primary NSCLC with different metastatic sites. We have identified critical receptor–ligand pairs that mediate the crosstalk between tumour cells and components of the TME, ultimately contributing to bone or brain metastasis in NSCLC.

## AUTHOR CONTRIBUTIONS

Zhi‐Hong Zhang, Yue‐Yin Pan, Jing‐Wen Fang, and Chuang Guo conceived and supervised the study. Ke Xu supervised sample collection and clinical annotation with help from Yu‐Xia Zou, Huan‐Huan Zhang, Yue‐Nan Wang, Xue‐Ru Ren, Ye‐Hong Xu, Jia‐Jun Li, Hao Tang, Cheng He, Song Wei, Tian Tian, Lai‐Lin Li, Hui Zhou and Lin‐Juan Xu. Jia‐Xuan Yang and You‐Yang Zhou performed data analysis. Jing‐Wen Fang and Chuang Guo helped reviewing the paper and provided critical data interpretation. Ke Xu and Hao Wang wrote the manuscript with input from all authors. All of authors have read and approved the manuscript.

## CONFLICT OF INTERESTS STATEMENT

The authors declare no conflict of interests.

## ETHICAL APPROVAL

The study was approved by The Human Investigation and Ethical Committee of Anhui Provincial Cancer Hospital (The First Affiliated Hospital of USTC West District). The Approval Number is 105/2021. All participants provided written informed consent.

## Supporting information

Supporting Information

Supporting Information

Supporting Information

Supporting Information

Supporting Information

Supporting Information

Supporting Information

Supporting Information

Supporting Information

## Data Availability

The raw sequence data reported in this paper have been deposited in the Genome Sequence Archive (Genomics, Proteomics & Bioinformatics 2021) in National Genomics Data Center (Nucleic Acids Res 2022), China National Center for Bioinformation / Beijing Institute of Genomics, Chinese Academy of Sciences (GSA‐Human: HRA003859) that are publicly accessible at https://ngdc.cncb.ac.cn/gsa‐human/s/ZEr4vvt4.
