# Supplementary figures and images for: Distinct fibroblast subpopulations associated with bone, brain or intrapulmonary metastasis in advanced non‐small‐cell lung cancer
Source: Clin Transl Med. 2024 Mar 6;14(3):e1605. doi: 10.1002/ctm2.1605 (PMC10915739; doi:10.1002/ctm2.1605)

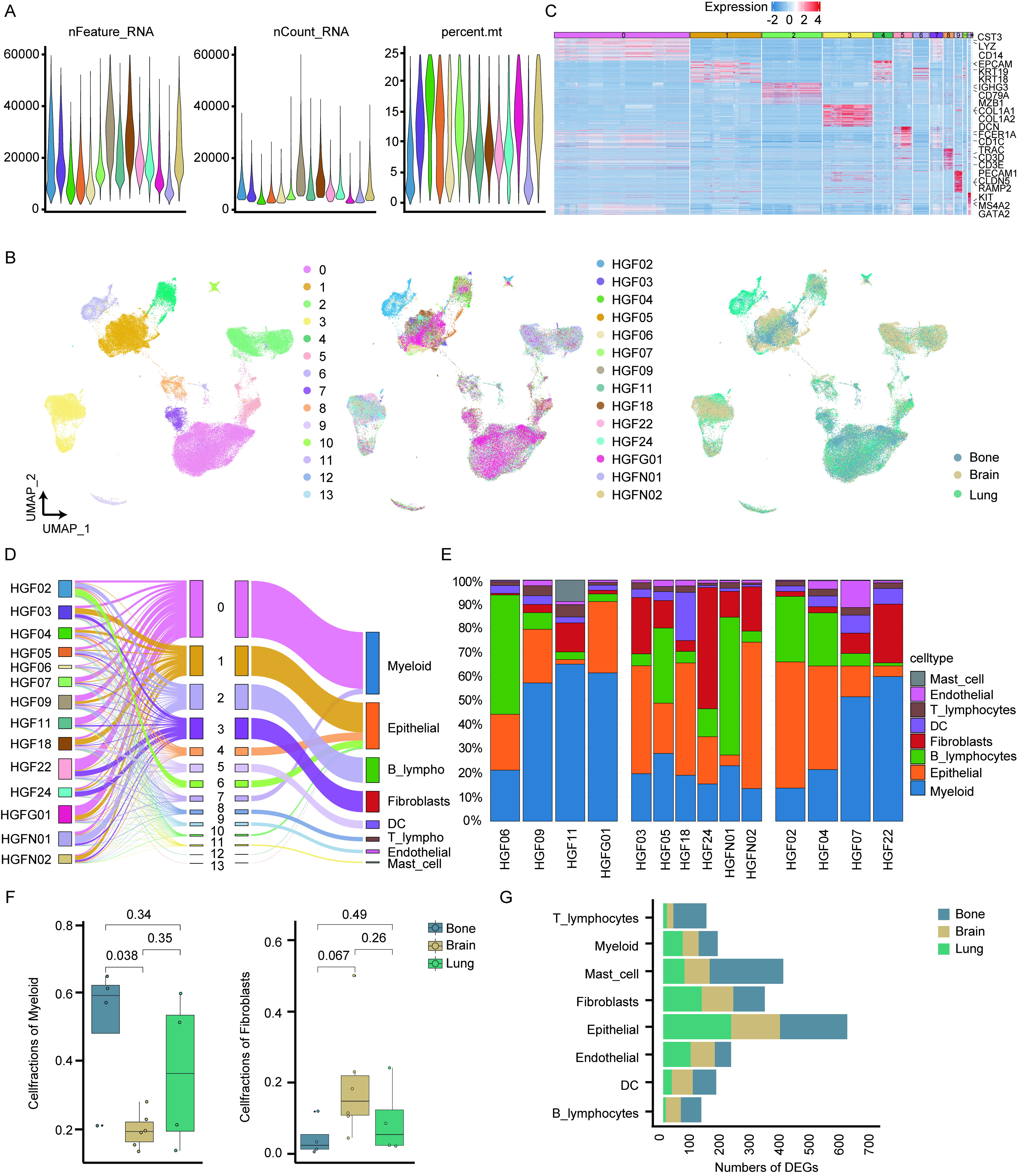

Supplement: Supplementary file 1 — Supporting Information [file CTM2-14-e1605-s008.tif]

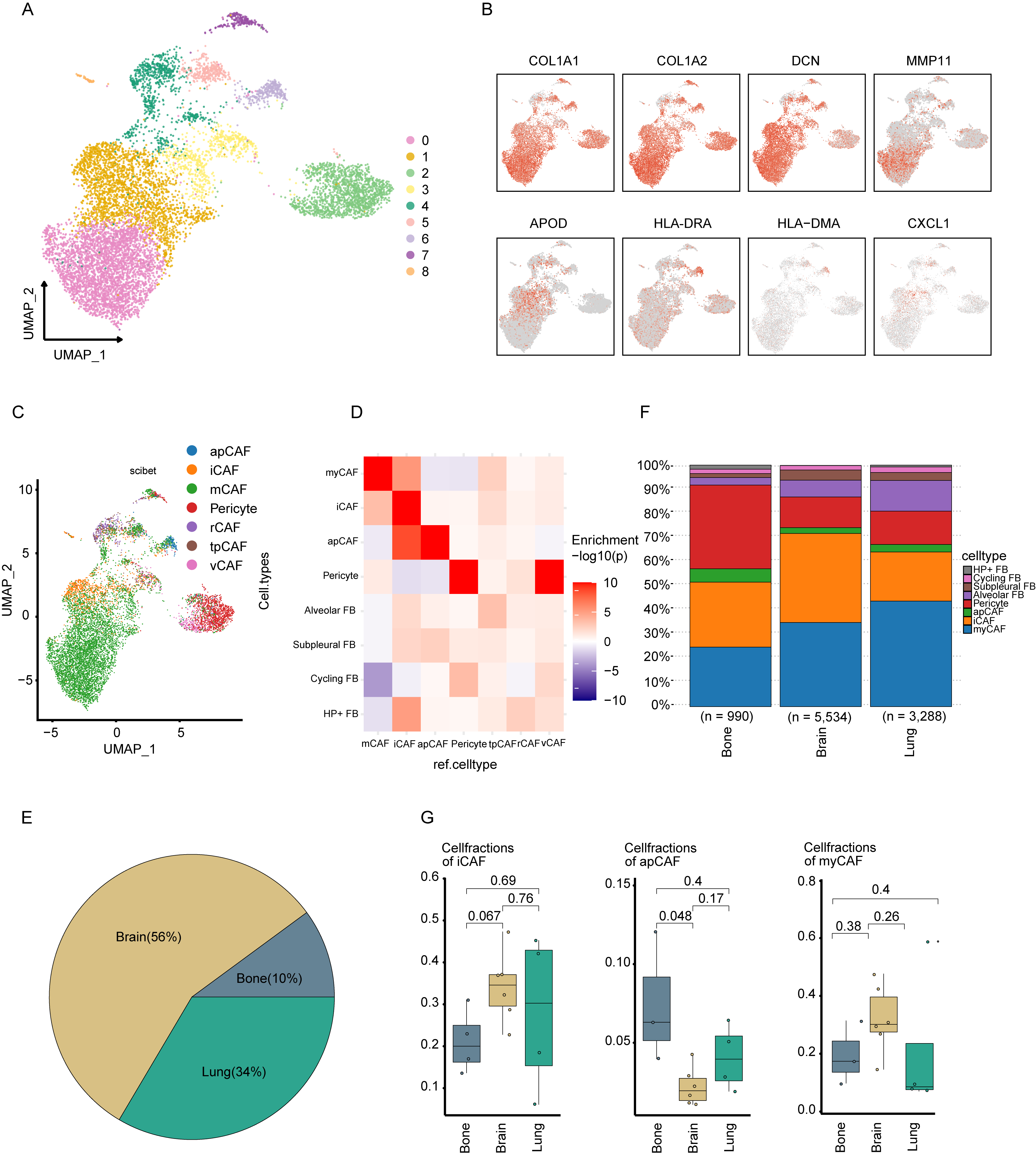

Supplement: Supplementary file 2 — Supporting Information [file CTM2-14-e1605-s001.tif]

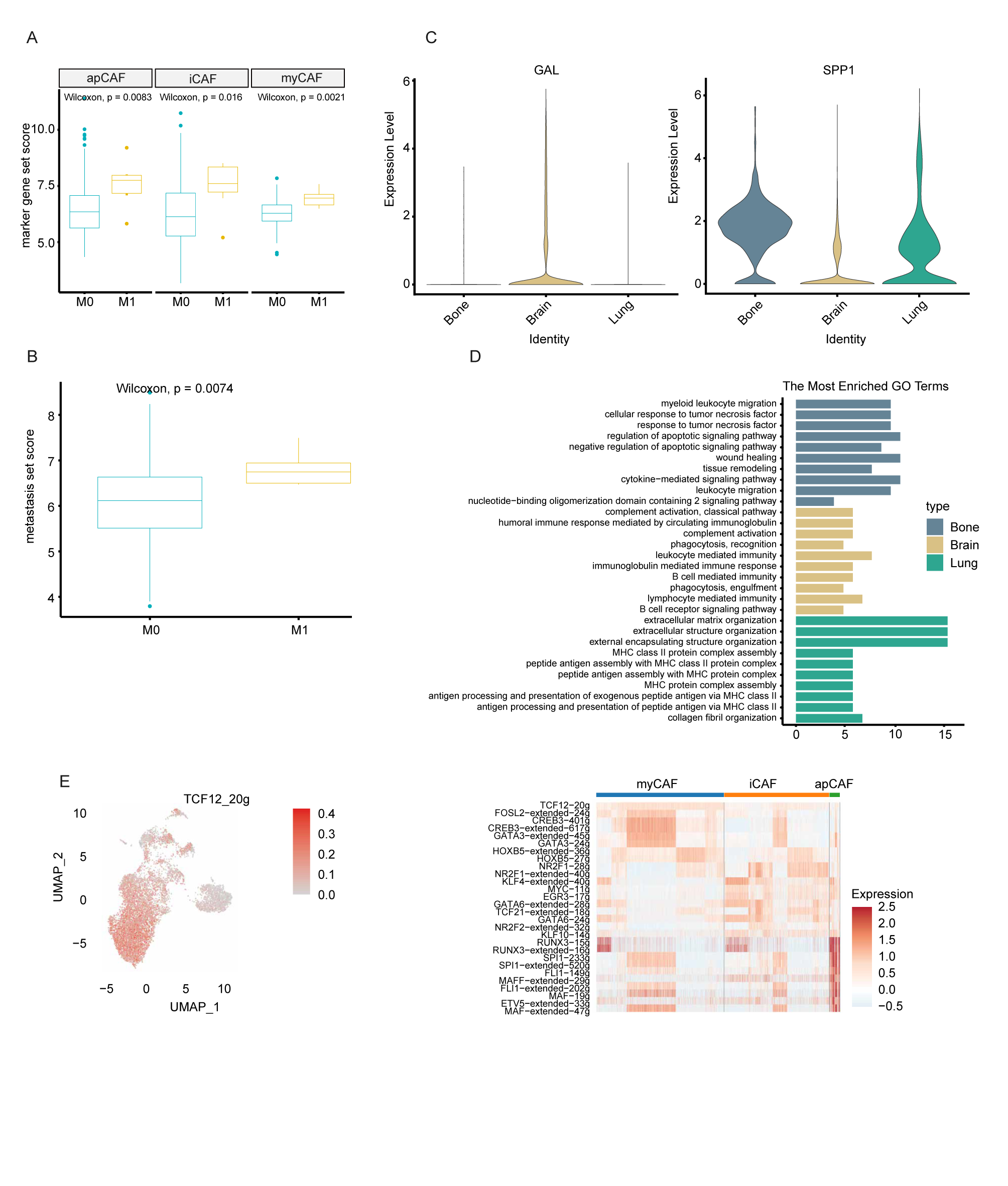

Supplement: Supplementary file 3 — Supporting Information [file CTM2-14-e1605-s007.tif]

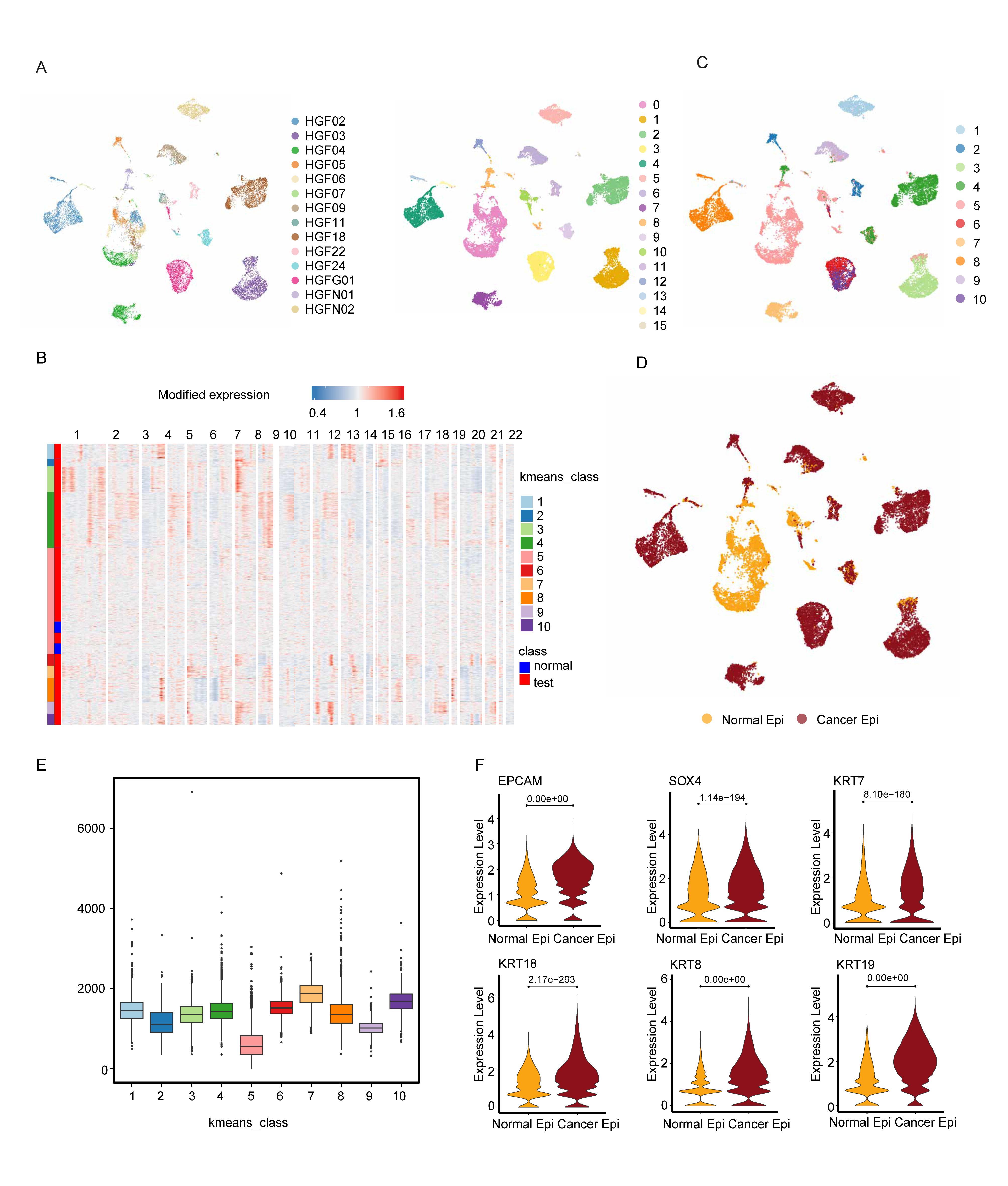

Supplement: Supplementary file 4 — Supporting Information [file CTM2-14-e1605-s006.tif]

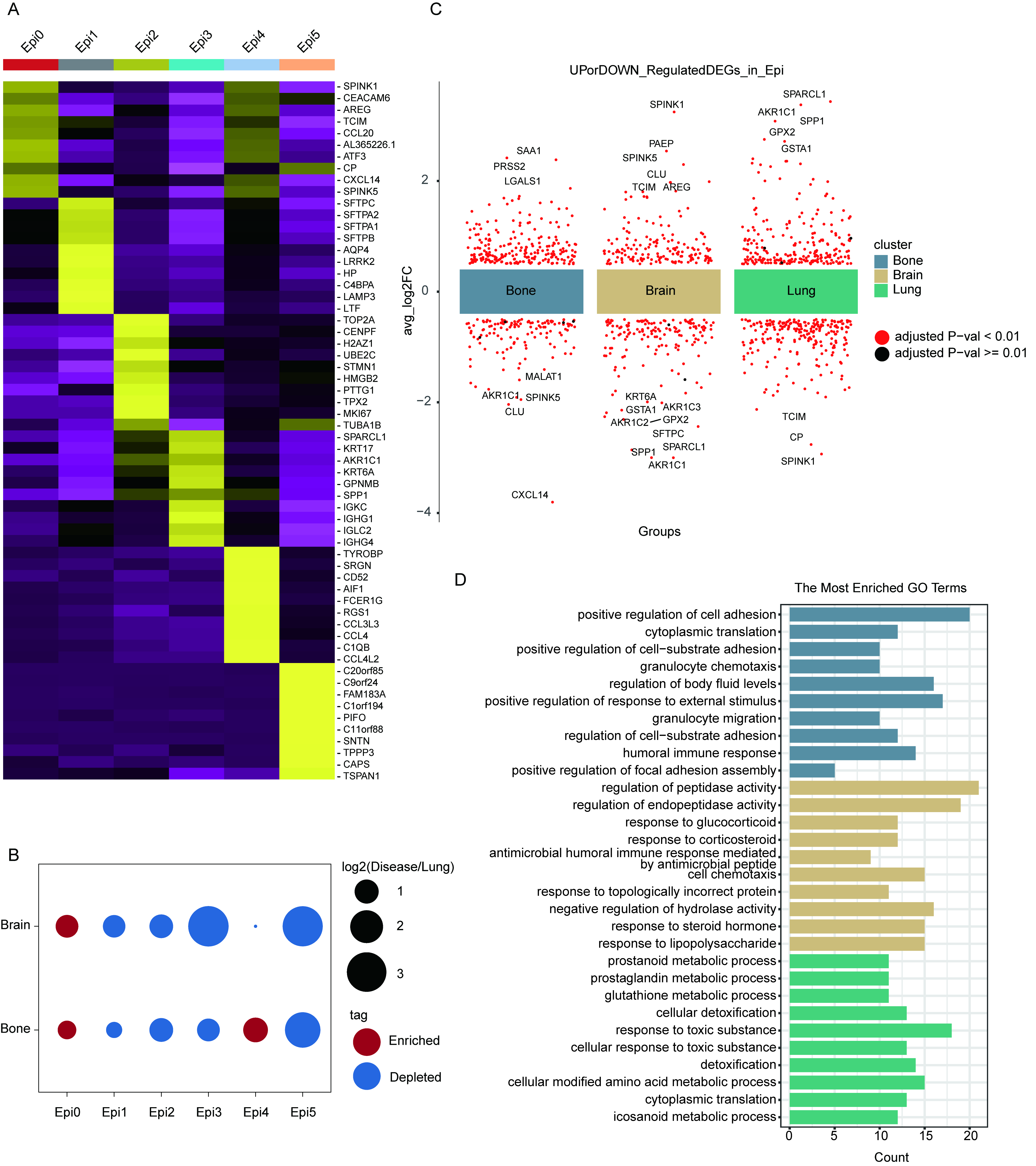

Supplement: Supplementary file 5 — Supporting Information [file CTM2-14-e1605-s009.tif]

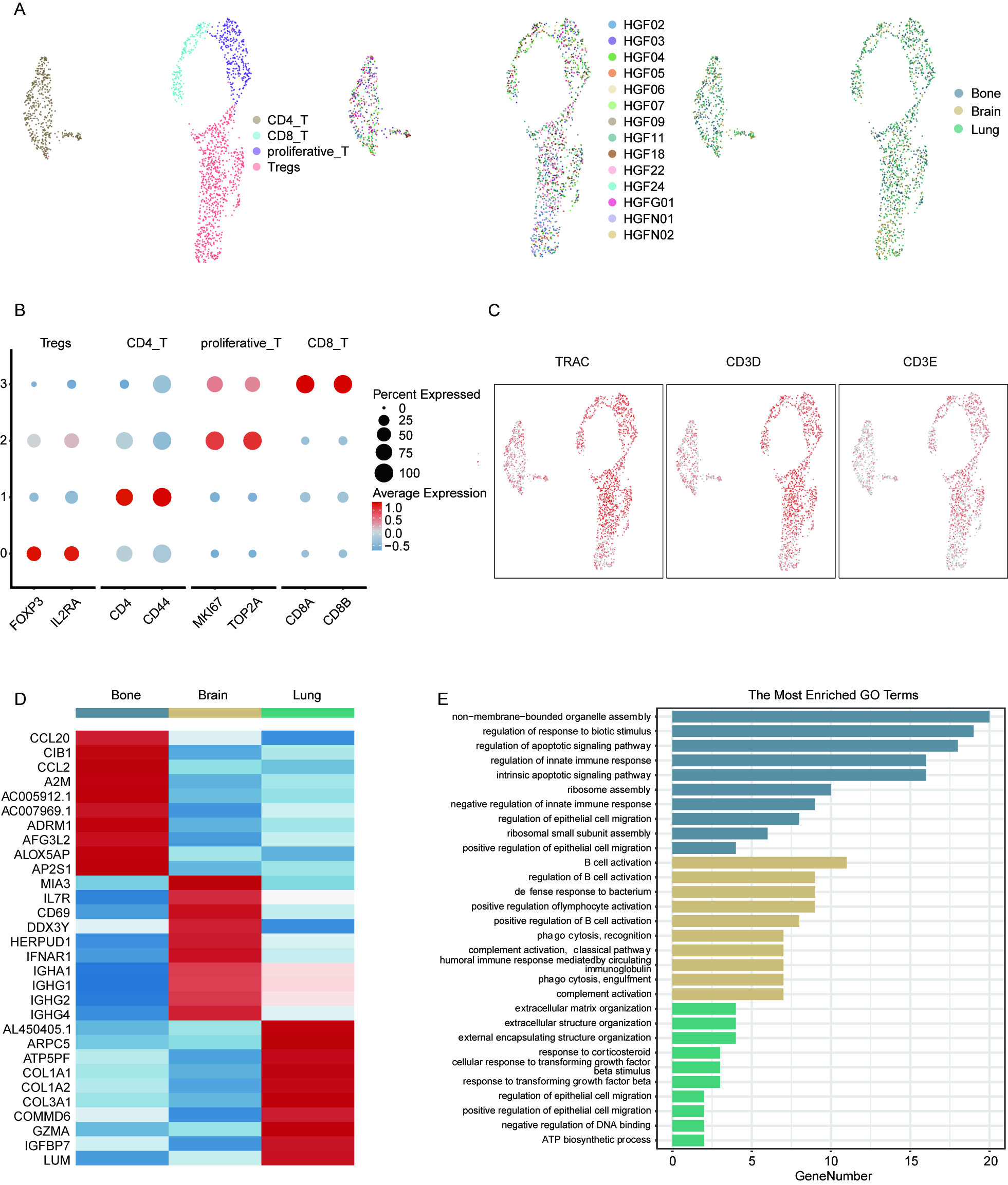

Supplement: Supplementary file 6 — Supporting Information [file CTM2-14-e1605-s004.tif]

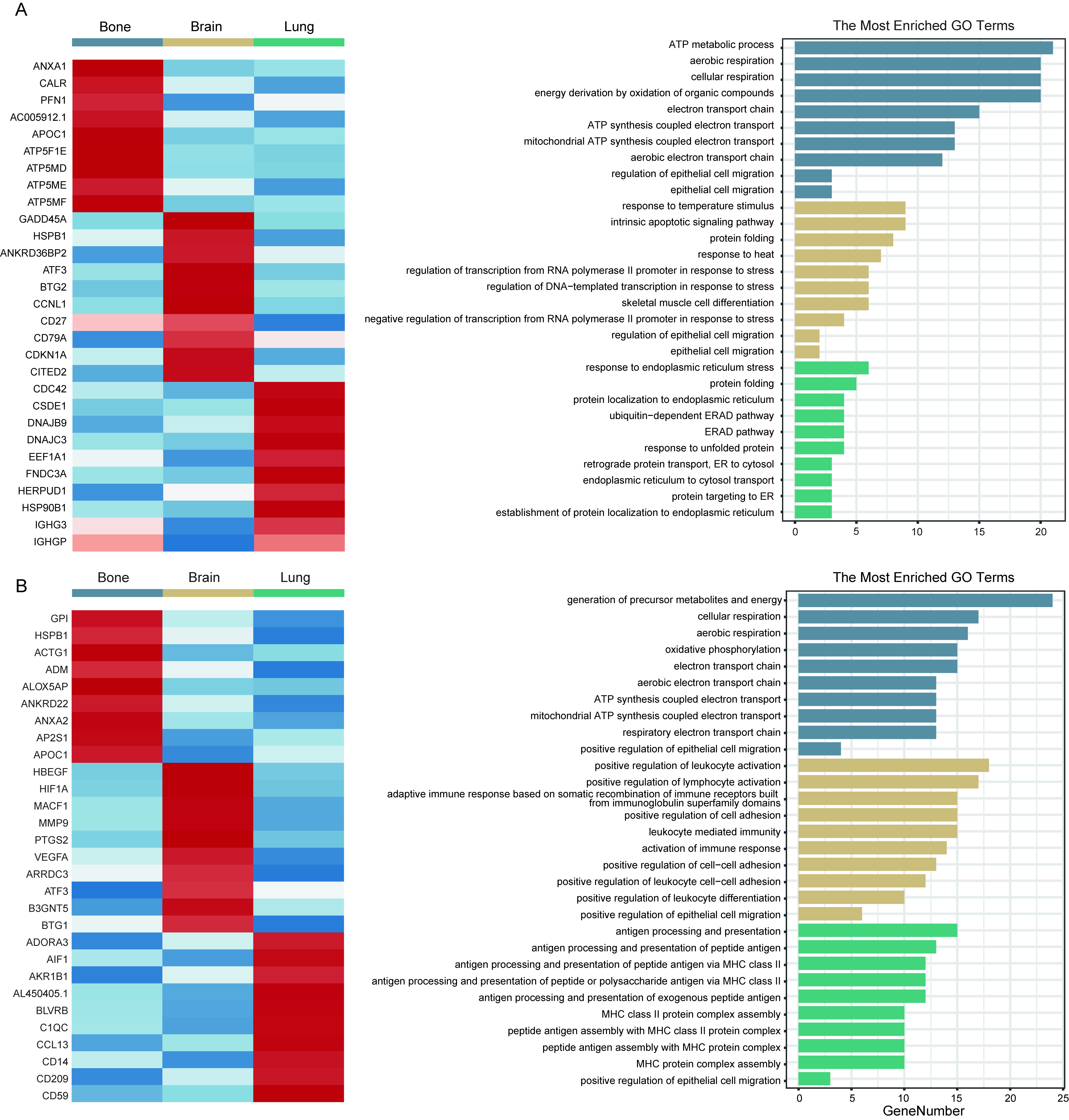

Supplement: Supplementary file 7 — Supporting Information [file CTM2-14-e1605-s005.tif]
